# Climate Warming May Facilitate Invasion of the Exotic Shrub *Lantana camara*

**DOI:** 10.1371/journal.pone.0105500

**Published:** 2014-09-03

**Authors:** Qiaoying Zhang, Yunchun Zhang, Shaolin Peng, Kristjan Zobel

**Affiliations:** 1 Department of Botany, Institute of Ecology and Earth Sciences, University of Tartu, Tartu, Estonia; 2 Department of Environmental Science and Engineering, Qilu University of Technology, Jinan, Shandong, China; 3 State Key Laboratory of Biocontrol, School of Life Sciences, Sun Yat-sen University, Guangzhou, Guangdong, China; University of New England, Australia

## Abstract

Plant species show different responses to the elevated temperatures that are resulting from global climate change, depending on their ecological and physiological characteristics. The highly invasive shrub *Lantana camara* occurs between the latitudes of 35°N and 35°S. According to current and future climate scenarios predicted by the CLIMEX model, climatically suitable areas for *L. camara* are projected to contract globally, despite expansions in some areas. The objective of this study was to test those predictions, using a pot experiment in which branch cuttings were grown at three different temperatures (22°C, 26°C and 30°C). We hypothesized that warming would facilitate the invasiveness of *L. camara*. In response to rising temperatures, the total biomass of *L. camara* did increase. Plants allocated more biomass to stems and enlarged their leaves more at 26°C and 30°C, which promoted light capture and assimilation. They did not appear to be stressed by higher temperatures, in fact photosynthesis and assimilation were enhanced. Using lettuce (*Lactuca sativa*) as a receptor plant in a bioassay experiment, we also tested the phytotoxicity of *L. camara* leachate at different temperatures. All aqueous extracts from fresh leaves significantly inhibited the germination and seedling growth of lettuce, and the allelopathic effects became stronger with increasing temperature. Our results provide key evidence that elevated temperature led to significant increases in growth along with physiological and allelopathic effects, which together indicate that global warming facilitates the invasion of *L. camara*.

## Introduction

Global average temperatures are increasing and are predicted to do so further in the future [1]. Changes in temperature and precipitation associated with rising concentrations of CO_2_ are altering local environmental conditions, which may inhibit native species [2], [3]. At the same time, this may provide some non-native species with emerging opportunities for population growth and expansions [4]. The successful invasion of new areas by non-native species can have serious ecological consequences for species interactions and ecosystem structure and functioning [5]. Therefore, it is essential to better understand the risk of invasion under climate change scenarios for effective management of invasive plants in the 21^st^ century [6].

The abundance and distribution of plant species are tightly regulated by both climatic factors [7] and biotic interactions [8], so changes in climatic conditions are likely to cause major shifts in their population dynamics and geographic ranges [2], [3], [9]. Apart from changes in the potential distributions of native species, climate change may also affect the spatial distribution of invasive species [5], [6], [9]. Previous studies have shown that global warming has enabled alien plants to expand into regions where they previously could not survive and reproduce [9]. Any alterations of plant community structure that are caused by climate change result from underlying changes in the population dynamics of species that make up the community [10]. Thus, understanding responses to climate change at the species level is important to the prediction of future ecosystem functioning [10].

The focal species of this study is *Lantana camara* (Verbenaceae), a small perennial shrub which can grow to around 2 m in height and forms dense thickets in a variety of environments [11]. Its native range is Central America, the northern part of South America and the Caribbean [11]. *L. camara* has been identified as one of the 100 World's Worst Invasive Alien Species [12]. Since the sixteenth century, it has been subject to intense horticultural improvement in Europe, and now it exists in many different forms and varieties around the world [13]. Its global distribution includes about 60 countries and islands between the latitudes of 35°N and 35°S [14]. *L. camara* has become a major problem in many of these areas, causing reductions in native species diversity, declines in soil fertility, allelopathic alteration of soil properties, and alteration of ecosystem processes [11], [14].

The model CLIMEX has been widely used to illustrate the potential distribution of species under future climate scenarios [15]. Based on CLIMEX simulations, the potential distribution of *L. camara* will expand in some areas under current and future climate scenarios [14], [16], [17], [18], and in China specifically its distribution could potentially expand further inland [11]. This is consistent with field investigations in southern China, where *L. camara* has recently become more prevalent [19]. Climate studies have shown that winter minimum temperatures in the region (i.e., Guangdong province) started to rise in the middle and later periods of the 1960s, and it has become warmer since the 1980s [20]. The observed increase in abundance is thus likely related to elevated temperature. We hypothesized that warming leads to positive effects on the fitness parameters of the invasive shrub *L. camara*, further facilitating its invasiveness.

We planted branch cuttings of *L. camara* in different temperature treatments (22, 26 and 30°C) in three experiments: a growth experiment, a physiological experiment and a bioassay experiment designed to assess the allelopathic potential of the species. Our goal was to describe and compare the morphological, physiological and biochemical responses of the species to future climate scenarios. Specifically, we sought to address the following questions: (1) How does the growth of *L. camara* respond to elevated temperatures? (2) How are gas exchange rates and photosynthesis affected by elevated temperatures? (3) Does the allelopathic potential of *L. camara* change with increasing temperature, as has been observed in some other plant species [21]?

## Methods

### Growth and morphology experiment

We collected three-year-old branches of *L. camara* on March 10, 2008 in Guangzhou, China. The sampling site (23°02′–23°04′N, 113°23′–113°24′E) was neither located on farmland nor in a protected area. No specific permissions were required for these locations/activities. No endangered or protected species were involved in the sampling. The tops of the branches were cut to keep them at least 20 cm long. The cuttings were planted at the experimental field of Sun Yat-sen University, Guangzhou, Guangdong province. After three weeks, we selected uniform branch cuttings and transplanted them into plastic pots (20-cm diameter, 15-cm height, with three branches per pot). The pots were filled with equal proportions of nutrient-rich soil and vermiculite for water retention. The plants were grown in different greenhouses (14/10 h day/night cycle, 75%±2% relative humidity, photosynthetically active radiation (PAR) 400 mmol m^−2^ s^−1^) at three constant temperatures (22, 26 and 30°C). There were 15 replicate pots per temperature treatment. All pots were randomly placed once a week to avoid internal effects. They were watered with diluted Hoagland solution (25% v/v) once a week, for a total of 18 weeks.

Then, at the end of the experiment, plants were harvested and divided into leaf blades, petioles, stems and roots, and were dried separately to a constant mass at 70°C. The total stem length was measured. Leaf area was determined using an LA meter (CI-203 Area-meter, CID, USA). The raw data were used to calculate the following growth parameters [22]: leaf mass ratio (leaves without petioles, LMR), root mass ratio (RMR), support organ mass ratio (stems and petioles, SMR), and specific leaf area (SLA). The mass ratio was calculated by dividing the dry mass by total plant dry mass.

### Physiological experiment

#### Gas exchange

After potted plants had been allowed to grow for 18 weeks at different temperatures, we measured the gas exchange of *L. camara* on fully expanded leaves under controlled optimal conditions, using an open system with a portable photosynthesis measurement system (LI-6400, LI-COR, USA). The measurements were made on 15 plants per treatment. We found out that under greenhouse conditions, the net photosynthetic rate (Pnet) of *L. camara* was greatest at 800–1200 µmol m^−2^ s^−1^, so we maintained PAR at 1200 µmol m^−2^ s^−1^. We used an LI-6400 artificial light source and maintained the temperature at 22, 26 and 30°C for each treatment. In order to avoid the effect of midday photosynthetic depression [23], we completed the measurements on two sunny days from 08:00 to 11:30 and from 15:00 to 17:30. Pnet and stomatal conductance (Cond) were also measured, while intrinsic water use efficiency (WUE) was calculated by dividing Pnet by Cond [23].

#### Chlorophyll (Chl) fluorescence

We used the saturation pulse method [24] to measure the Chl fluorescence. Measurements were taken from the upper surface of the same leaves used in the previously described measurements, with a pulse-amplitude-modulated fluorometer (PAM 2100, Walz, Effeltrich, Germany) [25]. Before measurement, the leaves were placed in dark for at least 30 min. The intensity and duration of the saturation pulses was 4,000 µmol m^−2^ s^−1^ and 0.8 s, respectively. The “actinic light” was 600 µmol m^−2^ s^−1^. We recorded the fluorescence parameters Fv/Fm and ΦPSII. Fv/Fm is the maximum quantum yield of photosystem II (PSII), which is assessed as (Fm - Fo)/Fm [26], where Fo and Fm are the minimal and maximal fluorescence values of a dark-adapted sample, respectively, with all of the PSII reaction centers fully open. It was measured at predawn, when plants were in the dark, to make sure that all the PSII reaction centers were open. ΦPSII is the effective quantum yield of PSII. It was calculated as ΦPSII = (Fm′−Ft)/Fm′, where Fm′ is the maximal fluorescence value reached in a pulse of saturating light with an illuminated sample, and Ft is the fluorescence value of the leaf at a given photosynthetically active radiation [27].

### Bioassay experiment on allelopathic potential

After the potted *L. camara* plants had been growing for 18 weeks at three different temperatures (22, 26 and 30°C), we collected fresh leaves (10 g) randomly from plants in each greenhouse and soaked them in distilled water (100 mL) for 24 h in darkness at 22, 26 and 30°C, respectively. We then made aqueous leachates with a concentration of 0.1 g mL^−1^ from each treatment. The pH value of all leachates was adjusted to 6.8 using 1 M NaOH or HCl, and distilled water was used as a control.

Twenty uniform lettuce (*Lactuca sativa*) seeds were selected, surface-sterilised with 0.5% KMnO_4_ for 15 min, and then washed with sterile water. The seeds were put on top of two layers of filter paper (9-cm diameter) in a glass Petri dish (9-cm diameter). Each dish contained 5 mL of aqueous leachate obtained from *L. camara* plants grown at different temperatures, while the controls contained 5 mL distilled water. The Petri dishes were kept in dark conditions at room temperature (∼22°C). All of the treatments were conducted with four independent replicates. The germinated seeds (once radicle length was about 1–2 mm) were counted every 12 h for the first day, and every 24 h thereafter. Germination of *L. sativa* was recorded up to 5 days, and seedling growth (root length and shoot length) was recorded at the end of the experiment, on the seventh day. The effect of the leachate on lettuce growth was evaluated using a response index (RI) [28] as follows:
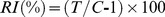
(1)where C was the control value and T was the treatment value. RI>0 indicates a stimulatory effect, while RI<0 indicates an inhibitory effect.

### Statistical analysis

The effect of different temperatures on growth, physiology and allelopathy of *L. camara* was assessed by one-way ANOVA and means were compared by Tukey tests. All statistical analyses were performed using the software R 3.0.1 [29].

## Results

### Growth and morphology

**Figure 1:**
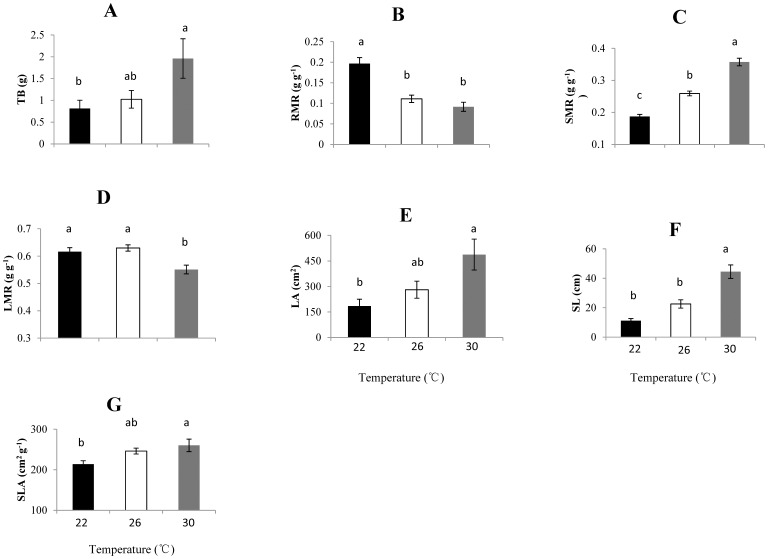
Responses of *Lantana camara* plants to temperature treatments (mean ± SE, *n* = 15). Different letters indicate significant differences (P<0.05) between means according to Tukey's HSD tests (The same below). (A) TB, total biomass; (B) RMR, root mass ratio; (C) SMR, support organ mass ratio; (D) LMR, leaf mass ratio; (E) LA, leaf area; (F) SL, stem length; (G) SLA, specific leaf area.

The overall biomass of *L. camara* plants, and their allocation of biomass to support organs (stem and petiole), were significantly higher in high-temperature treatments than in the control (22°C), while biomass allocation to roots (RMR) and leaves (LMR) displayed the opposite pattern (Table 1, Fig. 1). The leaf area, stem length and SLA of *L. camara* were also significantly higher at the elevated temperatures (Table 1, Fig. 1). The stem length of plants growing at 30°C was four times that of those at 22°C, while their leaf area was double. SLA at the elevated temperature of 30°C was 22.1% higher than at 22°C.

**Table 1:** F-values of one-way ANOVA which was used to test the effects of different temperatures on growth and morphology of *L. camara*.

	TB	RMR	SMR	LMR	LA	SL	SLA
d.f.	2, 16	2, 16	2, 16	2, 16	2, 16	2, 16	2, 16
F	4.04	22.47	91.03	8.92	5.76	27.00	4.70*
P	0.038*	<0.001***	<0.001***	0.003**	0.013*	<0.001***	0.025*

*** *P*<0.001,

** P<0.01,

* P<0.05.

Parameters of growth and morphology:

TB: total biomass; RMR: root mass ratio; SMR: support organ mass ratio; LMR: leaf mass ratio; LA: leaf area; SL: stem length; SLA: specific leaf area.

### Photosynthesis and chlorophyll fluorescence

#### Gas exchange

**Figure 2:**
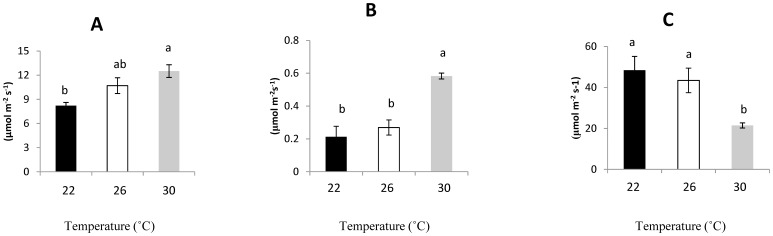
Gas exchange of *L. camara* seedlings growing at different temperatures. Data are means ± SE (n = 15). (A) Pnet, Net photosynthetic rate; (B) Cond, stomatal conductance; (C) WUE, water use efficiency.

Plants growing at 30°C showed significantly higher Pnet and Cond, yet lower WUE than those growing at 22°C (Table 2, Fig. 2 A–C). No significant differences in any gas exchange parameters were found between seedlings growing at 22°C and 26°C, while seedlings growing at 26°C exhibited significantly lower Cond and higher WUE than those growing at 30°C (Table 2, Fig. 2 B, C).

**Table 2:** F-values of one-way ANOVA which was used to test the effects of different temperatures on gas exchange and chlorophyll fluorescence, and allelopathic potential of *L. camara*.

	Pnet	Cond	WUE	Fv/Fm	ΦPSII	Germ	SH	RL
d.f.	2, 16	2, 16	2, 16	2, 16	2, 16	2, 16	2, 16	2,16
F	9.047	15.61	6.63	0.627	0.452	0.756	7.893	9.608
P	0.0035*	<0.001***	0.010*	0.547	0.645	0.497	0.001**	<0.001***

*** *P*<0.001,

** P<0.01,

* P<0.05.

Parameters of gas exchange and chlorophyll fluorescence:

Pnet: net photosynthetic rate; Cond: stomatal conductance; WUE: intrinsic water use efficiency; Fv/Fm; ΦPSII.

Parameters of allelopathic potential: Germ: germination; SH: shoot length; RL: root length.

#### Chlorophyll fluorescence

**Figure 3:**
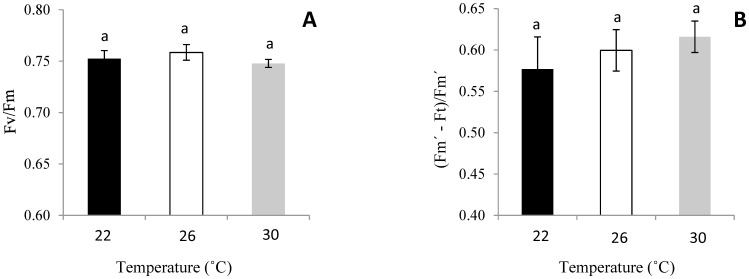
Chlorophyll fluorescence of *L. camara* seedlings growing at different temperatures. Data is means ±SE (n = 15). (A) Fv/Fm; (B) ΦPSII.

No significant differences in Fv/Fm and ΦPSII were found among seedlings of *L. camara* growing at the three different temperatures (Table 2, Fig. 3 A, B).

### Allelopathic potential

**Figure 4:**
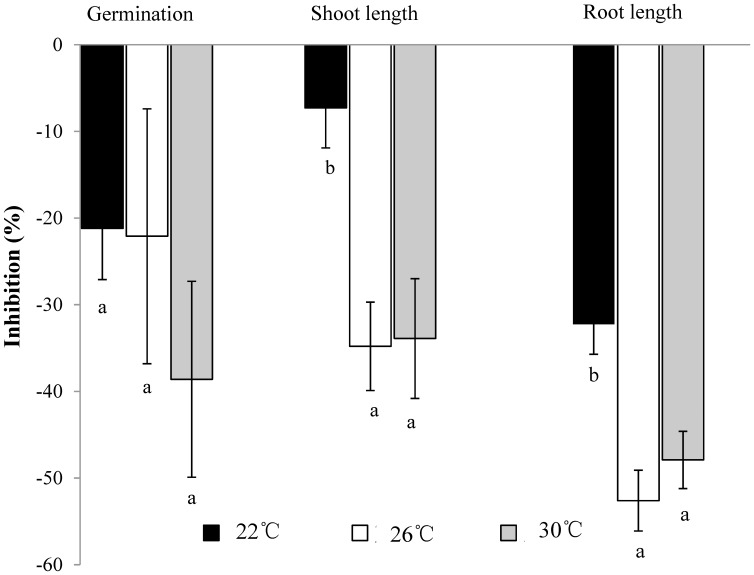
Allelopathic effects of aqueous leachate from fresh leaves of *L. camara* on seed germination, shoot length and root length of *Lactuca sativa*, expressed as a response index at different temperatures. Each bar represents a mean ± SE.

The allelopathic effects of aqueous leachate from *L. camara* leaves on seed germination and seedling development of lettuce at different temperatures were evaluated (Table 2, Fig. 4). The shoot and root length of lettuce significantly decreased with an increase in temperature, but there were no significant differences in germination between different temperatures. The allelopathic effects on shoot and root length were significantly greater at the higher temperatures compared to at 22°C, with the highest effect occurring at 26°C.

## Discussion

Over the course of human history, people have intentionally or unintentionally moved innumerable plant species outside of their native ranges, and many of those alien plants become invasive [6]. Human activities are also partly responsible for the increase of global surface temperatures [21], [30], [31]. A number of recent studies on invasive plants and climate change have shown that increasing temperatures and changing precipitation might either “help” or “hinder” invasive plants, depending on the species, location and dominant forces causing changes in climate conditions [6], [9], [21], [32]. Such variation makes it challenging to assess and understand the mechanisms that might facilitate or constrain the success of invasive species in the context of climate change [5].

As our findings illustrate, climate warming affects many aspects of the invasive species *L. camara*'s biology and ecology. Firstly, elevated temperature caused changes in the biomass allocation and morphology of plants. Plant growth is directly influenced by biomass allocation between leaves, stems, and other plant parts [33]. With rising temperature, individuals exhibited a significant increase in stem length, and biomass allocation to stems and petioles at the expense of leaves and roots. These changes may ensure greater structural support and an increased ability to capture light. Although biomass allocation to leaves decreased as in temperature increased, SLA and LA were greatest in the high-temperature treatments. SLA is a plant trait that is important for the regulation and control of functions such as carbon assimilation and carbon allocation [34], [35]. Generally, the combination of increased SLA and LA results in increased light absorption, and shading of other species [36]. This may be a light utilization strategy that could enhance the competitive ability of *L. camara*, because this species cannot survive under the dense, continuous canopies of taller native forest species due to the lack of light [14]. *L. camara* usually flowers in the first growing season after its establishment and, if adequate moisture and light are available, it can flower in all seasons [37]. If the mean global temperature will rise by 1.4–5.8°C over the period of 1990–2100 as predicted [30], *L. camara* may increase in height more rapidly than its neighbours, and then suppress their growth by shading them. It may also flower more often and for longer periods of time, enabling it to produce more offspring, which could cause substantial damage to other species and their ecosystems. The responses to the increase in temperature observed in our study suggest that warming may help *L. camara* to reach further into the upper layer of the plant community, and expand its leaves as much as possible for better light capture and assimilation so as to facilitate its invasion.

Secondly, elevated temperature induced changes in the physiological parameters of *L. camara*. We found that plants exhibited a significantly higher photosynthetic capacity at higher temperatures (26 and 30°C) than at 22°C, which may be ascribed to a higher Pnet. High temperatures tended to increase stomatal conductance (Fig. 2 B, [38]), which can augment water loss. Consequently, the instantaneous water-use efficiency (WUE) of plants decreased with increasing temperature (Fig. 2 C). This response may explain why *L. camara* mostly invades wetter habitats [14]. High temperatures can influence photosynthesis in different ways, such as enhancing membrane fluidity and oxidative stress [38], or by changing the activity of the Calvin cycle and photorespiration [39]. High temperature may also inhibit the repair of PSII [40]. In this study, there were no significant differences in Fv/Fm and ΦPSII among different temperature conditions, and *L. camara* showed optimal functioning of its PSII with very low photoinhibition levels (*F*v/*F*m from ∼0.750 to 0.870) after exposure to higher temperatures. This suggested that higher temperature did not lead to stress in *L. camara*. Higher photosynthesis can increase invasive plants' growth rates and biomass accumulation, which may enable invasive species to out-compete slower growing species and hence facilitate their colonization [41], [42]. The responses we observed to elevated temperatures can be viewed as positive effects of warming on the physiological parameters of *L. camara*, i.e., increased rates of photosynthesis at higher temperatures could facilitate its invasive success. Of course, this enhancement of the plant's growth is often a “negative” effect at the ecosystem level.

Thirdly, elevated temperature induced changes in the allelopathic effects of *L. camara*. Those effects have been well-documented to cause severely reduced seedling recruitment in almost all species exposed to *L. camara*, and a reduction in the DBH growth of mature trees and shrubs [43], [44]. The allelopathic effects of *L. camara* may explain why it can survive secondary succession and form monospecific thickets [14]. In our experiment, we found that its phytotoxicity increased with temperature, with respect to both seed germination and seedling growth of the receptor plants (Table 2, Fig. 4). This result is consistent with other research about how elevated temperature influences the allelopathic effects of invasive plants [21], [45]. Allelopathic biochemicals produced by invasive plants function as their “novel weapons” since they can inhibit the growth of native plants in the invaded communities [46]. The increased phytotoxicity of *L. camara* in higher temperatures may be a result of the plant producing more allelochemicals, or of its allelochemicals becoming more phytotoxic under elevated temperatures [21]. As such, we can conclude that warming also enhanced the allelopathic potential of *L. camara*.

Biological invasions and climate change are key factors that are currently affecting global biodiversity [9] and the relationship between them is very complex [6]. In this study, we chose to study temperature, one of the most important elements of climate change, to understand its effects on plant invasion. The results showed that elevated temperature resulted in significant increases in biomass allocation and beneficial changes in morphology, photosynthesis and allelopathic effects of *L. camara*, indicating that global warming could facilitate the invasion of this plant. Based on the predictions of climate models, a 1°C increase in mean annual temperature could result in a pole-ward shift of each of the world's vegetation zones by approximately 200 km [2]. If the global temperature increases by 1.4–5.8°C as predicted [30], *L. camara* will likely migrate toward higher latitudes.

Our experiment was conducted in the absence of competition from surrounding plants. In fact, competition with surrounding native species is one of the most important factors that influence the outcomes of invasion by alien plants. Under future climate scenarios, both native and invasive species are likely to grow more vigorously [42], which could affect competitive interactions in the invaded habitats. Future studies should also address the biotic factors that affect the invasiveness of *L. camara*.

## References

[1] IPCC (2007) Climate Change 2007: Synthesis Report. Summary for Policymakers. Intergovernmental Panel on Climate Change, Cambridge University Press.

[2] Walther GR, Post E, Convey P, Menzel A, Parmesan C, et al (2002) Ecological responses to recent climate change. Nature 416: 389–395 10.1038/416389a 11919621

[3] Parmesan C (2006) Ecological and evolutionary responses to recent climate change. Annual Review of Ecology, Evolution, and Systematics 37: 637–669.

[4] Sorte CJB, Ibàñez I, Blumenthal DM, Molinari NA, Miller LP, et al (2013) Poised to prosper? A cross-system comparison of climate change effects on native and non-native species performance. Ecology Letters 16 2: 261–270 10.1111/ele.12017 23062213

[5] Weitere M, Vohmann A, Schulz N, Linn C, Dietrich D, et al (2009) Linking environmental warming to the fitness of the invasive clam *Corbicula fluminea* . Global Change Biology 15: 2838–2851 10.1111/j.1365-2486.2009.01925.x

[6] Bradley BA, Blumenthal DM, Wilcove DS, Ziska LH (2010) Predicting plant invasions in an era of global change. Trends in Ecology & Evolution 25 5: 310–318. 20097441 10.1016/j.tree.2009.12.003

[7] Woodward FI (1987) Climate and Plant Distribution. Cambridge University Press.

[8] Araújo MB, Luoto M (2007) The importance of biotic interactions for modelling species distributions under climate change. Global Ecology and Biogeography 16 6: 743–753.

[9] Walther GR, Roques A, Hulme PE, Sykes MT, Pysek P, et al (2009) Alien species in a warmer world: risks and opportunities. Trends in Ecology and Evolution 24 12: 686–693. 19712994 10.1016/j.tree.2009.06.008

[10] Williams AL, Wills KE, Janes JK, Vander Schoor JK, Newton PC, et al (2007) Warming and free-air CO_2_ enrichment alter demographics in four co-occurring grassland species. New Phytologist 176: 365–374. 17888117 10.1111/j.1469-8137.2007.02170.x

[11] Taylor S, Kumar L, Reid N, Kriticos DJ (2012) Climate Change and the Potential Distribution of an Invasive Shrub, *Lantana camara* L. PLoS ONE 7 4: e35565 10.1371/journal.pone.0035565 22536408 PMC3334920

[12] Invasive Species Specialist Group, IUCN (2001) 100 World's Worst Invasive Alien Species. Available: http://www.issg.org/database/species/reference_files/100 English.pdf

[13] Thomas SE, Ellison CA (2000) A Century of Classical Biological Control of *Lantana camara*: Can pathogens make a significant difference? pp. 97–104. In: Spencer, N.R. [ed.], Proceedings of the X International Symposium on Biological Control of Weeds, Bozeman, USA, 1999.

[14] Day MD, Wiley CJ, Playford J, Zalucki MP (2003) Lantana: Current management Status and Future Prospects. ACIAR Monograph series, Canberra.

[15] Shabani F, Kumar L, Taylor S (2012) Climate change impacts on the future distribution of Date Palms: A modelling exercise using CLIMEX. PLoS ONE 7 10: e48021 10.1371/journal.pone.0048021 23110162 PMC3480471

[16] Taylor S, Kumar L (2013) Potential distribution of an invasive species under climate change scenarios using CLIMEX and soil drainage: A case study of *Lantana camara* L. in Queensland, Australia. Journal of Environmental Management 114: 414–422. 23164541 10.1016/j.jenvman.2012.10.039

[17] Taylor S, Kumar L (2012) Sensitivity analysis of CLIMEX parameters in modelling potential distribution of *Lantana camara* L. PLoS ONE 7 7: e40969 10.1371/journal.pone.0040969 22815881 PMC3398004

[18] Taylor S, Kumar L, Reid N (2012) Impacts of climate change and land-use on the potential distribution of an invasive weed: a case study of *Lantana camara* in Australia. Weed Research 52: 391–401.

[19] Shan JL (2003) Preliminary studies on exotic plant communities in Hainan. Chinese Journal of tropical agriculture 23 3: 1–4;51.

[20] Liang JY, Wu SS (2000) Climatological diagnosis of winter temperature variations in Guangdong. Journal of Tropical Meteorology 6: 37–45.

[21] Wang RL, Zeng RS, Peng SL, Chen BM, Liang XT, et al (2011) Elevated temperature may accelerate invasive expansion of the liana plant *Ipomoea cairica* . Weed research 51: 574–580.

[22] Poorter L (1999) Growth responses of 15 rain-forest tree species to a light gradient: the relative importance of morphological and physiological traits. Functional Ecology 13 3: 396–410 10.1046/j.1365-2435.1999.00332.x

[23] Zhang YC, Zhang QY, Luo P, Wu N (2009) Photosynthetic response of *Fragaria orientalis* in different water contrast clonal integration. Ecological Research 24 3: 617–625.

[24] Schreiber U, Bilger W, Hormann H, Neubauer C (1998) Chlorophyll fluorescence as a diagnostic tool: basics and some aspects of practical relevance. In: Photosynthesis: A Comprehensive Treatise (ed. A. S. Raghavendra) pp. 320–336. Cambridge University Press, Cambridge.

[25] Brugnoli E, Björkman O (1992) Chloroplast movements in leaves - influence on chlorophyll fluorescence and measurements of light-induced absorbency changes related to Delta-Ph and Zeaxanthin formation. Photosynthesis Research 32: 23–35. 24408152 10.1007/BF00028795

[26] Bolhar-Nordenkampf HR, Long SP, Baker NR, Oquist G, Schreiber U, et al (1989) Chlorophyll fluorescence as a probe of the photosynthetic competence of leaves in the field: A review of current instrumentation. Functional Ecology 3: 497–514.

[27] Genty B, Briantais JM, Baker NR (1989) The relationship between the quantum yield of photosynthetic electron transport and quenching of chlorophyll fluorescence. Biochimica et Biophysica Acta 990: 87–92.

[28] Williamson GB, Richardson D (1988) Bioassays for allelopathy: measuring treatment responses within dependent control. Journal of Chemical Ecology 14 1: 181–187. 24277003 10.1007/BF01022540

[29] R Development Core Team (2013) R: A Language and Environment for Statistical Computing. R Foundation for Statistical Computing, Vienna, Austria. ISBN 3-900051-07-0, URL http://www.R-project.org

[30] Houghton JT, Ding Y, Griggs DJ, Noguer M, van der Linden PJ, et al. (2001) The projections of the earth's future climate. In: Climate Change 2001: The Scientific Basis (eds JT Houghton, Y Ding, DJ Griggs, M Noguer, PJ Van Der Linden et al.), 62–77. Cambridge University Press, Cambridge, UK.

[31] Li MH, Kräuchi N, Gao SP (2006) Global warming: can existing reserves really preserve current levels of biological diversity? Journal of Integrative Plant Biology 48: 255–259.

[32] Bradley BA, Oppenheimer M, Wilcove DS (2009) Climate change and plant invasion: restoration opportunities ahead? Global Change Biology 15: 1511–152.

[33] Reich PB, Tjoelker MG, Walters MB, Vanderklein DW, Buschena C (1998) Close association of RGR, leaf and root morphology, seed mass and shade tolerance in seedlings of nine boreal tree species grown in high and low light. Functional Ecology 12 3: 327–338 10.1046/j.1365-2435.1998.00208.x

[34] Reich PB, Walters MB, Ellsworth DS (1997) From tropics to tundra: global convergence in plant functioning. Proc Natl Acad Sci USA 94 25: 13730–13734 10.1073/pnas.94.25.13730. PMID:9391094 9391094 PMC28374

[35] Lambers H, Poorter H (2004) Inherent variation in growth rate between higher plants: a search for physiological causes and ecological consequences. Academic Press, London, New York.

[36] Qin Z, Mao DJ, Quan GM, Zhang JE, Xie JF, et al (2012) Physiological and morphological responses of invasive *Ambrosia artemisiifolia* (common ragweed) to different irradiances. Botany 90: 1284–1294.

[37] Sharma GP, Raghubanshi AS, Singh JS (2005) Lantana invasion: an overview. Weed Biology and Management 5: 157–167.

[38] Carrion-Tacuri J, Rubio-Casal AE, de Cires A, Figueroa ME, Castillo JM (2013) Effect of low and high temperatures on the photosynthetic performance of *Lantana camara* L. leaves in darkness. Russian Journal of Plant Physiology 60 3: 322–329.

[39] Yordanov I (1992) Response of Photosynthetic Apparatus to Temperature Stress and Molecular Mechanisms of Its Adaptation. Photosynthetica 26: 517–531.

[40] Allakhverdiev SI, Kreslavskii VD, Klimov VV, Los DA, Carpentier R, et al (2008) Heat stress: An overview of molecular responses in photosynthesis. Photosynthesis Research 98: 541–550. 18649006 10.1007/s11120-008-9331-0

[41] Lambers H, Poorter H (1992) Inherent variation in growth rate between higher plants: A search for physiological causes and ecological consequences. Adv Ecol Res 23: 187–261.

[42] Anderson LJ, Cipollini D (2013) Gas exchange, growth, and defense responses of invasive *Alliaria petiolata* (Brassicaceae) and native *Geum vernum* (Rosaceae) to elevated atmospheric CO_2_ and warm spring temperatures. American Journal of Botany 100 8: 1544–1554. 23857735 10.3732/ajb.1300014

[43] Gentle CB, Duggin JD (1997) Allelopathy as a competitive strategy in persistent thickets of *Lantana camara* L. in three Australian forest communities. Plant Ecology 132: 85–95.

[44] Zhang QY, Peng SL, Zhang YC (2009) Allelopathic potential of reproductive organs of exotic weed *Lantana camara* . Allelopathy Journal 23: 213–220.

[45] Lobón NC, Gallego JC, Daz TS, Garcia JC (2002) Allelopathic potential of Cistus ladanifer chemicals in response to variations of light and temperature. Chemoecology 12: 139–145.

[46] Callaway RM, Aschehoug ET (2000) Invasive plants versus their new and old neighbors: A mechanism for exotic invasion. Science 290 5491: 521–523. 11039934 10.1126/science.290.5491.521

